# Clinical effectiveness of training for awareness, resilience, and action for adolescents and young adults with depression: The pilot phase of a multicenter randomized controlled trial

**DOI:** 10.3389/fpsyt.2023.1130035

**Published:** 2023-03-31

**Authors:** Erik Ekbäck, Lina Rådmark, Gabriel Granåsen, Rachel Svärling, Matilda Sörlin, Caspar Schönbeck, Eva Henje

**Affiliations:** ^1^Child and Adolescent Psychiatry, Department of Clinical Science, Umeå University, Umeå, Sweden; ^2^Department of Public Health and Clinical Medicine, Umeå University, Umeå, Sweden

**Keywords:** adolescents, young adults, depression, clinical trial, yoga, mindfulness, feasibility studies, online intervention

## Abstract

**Background:**

Depression is a top-ranking global health concern increasing in magnitude. Available treatments for adolescents and young adults are not convincingly effective and relapse rates remain high. Training for Awareness, Resilience and Action (TARA) is a group treatment program targeting specific pathophysiological mechanisms of depression in young people. TARA is feasible, acceptable, preliminarily efficacious in depressed American adolescents, and it affects postulated brain-circuitry.

**Methods:**

As an initial step of a multicenter randomized controlled trial (RCT) we performed a single-arm multicenter pilot-study on TARA. Thirty-five depressed individuals (15–21 years old, 28 females) received TARA for 12 weeks face-to-face or online. Data was collected before (T0), during, and after the intervention (T1). The trial was pre-registered at clinicaltrials.gov, NCT Registration: identifier [NCT04747340]. Feasibility outcomes included recruitment, attendance rates, and session ratings. Adverse events were recorded weekly and extracted from medical records at the end of the trial. Primary effectiveness outcome was self-rated depression severity on Reynolds Adolescent Depression scale 2nd ed. at T1. Secondary outcomes were Children’s Depression Rating Scale-revised (CDRS-R) and Multidimensional Anxiety Scale for Children (MASC) at T1.

**Results:**

TARA was feasible and safe in the present trial. No significant RADS-2-change was seen (adjusted mean difference –3.26, 95 % CI –8.35 to 1.83; *p*= 0.20), however a significant decrease in CDRS-R scores is reported (adjusted mean difference –9.99, 95% CI –14.76 to –5.22; *p* < 0.001). MASC-scores did not change significantly (adjusted mean difference 1.98, 95% CI –0.96 to 4.91; *p*=0.18). Additional feasibility aspects are presented and discussed.

**Discussion:**

Limitations include substantial loss-to-follow-up, no randomization to control, and that some participants received concomitant treatment(s). The Coronavirus pandemic complicated both implementation and interpretation of the trial. In conclusion TARA was feasible and safe in depressed adolescents and young adults. Preliminary signs of effectiveness were seen. The initiated RCT will be important and worthwhile to conduct, and several improvements to the design are suggested based on the present results.

**Clinical Trial Registration:**

clinicaltrials.gov, identifier NCT04747340.

## 1. Introduction

Major depressive disorder (MDD) is currently the leading cause of disability worldwide (1) and it is predicted by the World Health Organization that it will be the largest contributor to the global disease burden in 2030 (2). With early onset MDD there is a threefold increase in the lifetime risk of adult depressive episodes as recurrence rates remain high (3). By intervening effectively at an early age with strategies that may increase resilience and prevent relapse there is a potential to dramatically decrease the accumulated disease burden associated with MDD. However, there is limited evidence supporting the effectiveness of currently available interventions, such as cognitive behavioral therapy (CBT) and Selective Serotonin Reuptake Inhibitors (SSRI) for adolescents with MDD both in the acute phase (4) and in relapse prevention (5, 6). Cochrane reviewers have concluded that “on the basis of currently available evidence, the effectiveness of these interventions for treating depressive disorders in children and adolescents cannot be established” (7). Several subsequent meta-analyses have been consistent with the conclusion that the effectiveness of currently available interventions is questionable (4, 8–13), and that there is a need for treatment development and research to improve immediate and long term effects (13).

The theoretical basis for the current use of SSRI and CBT in adolescent MDD comes mainly from studies of adults, even though it is well known that the depressive neuro-psychopathology differs between adolescents and adults (14–17). Based on evidence of limbic hyperreactivity and reduced flexibility in the default mode network, as well as autonomic, and allostatic dysregulation in adolescent depression, we developed a novel group treatment program called “Training for Awareness, Resilience and Action” (TARA) (18). TARA was designed in a pragmatic manner targeting relevant domains and constructs of the Research Domain Criteria (RDoC) matrix (19). The RDoC-initiative supports the development of precision medicine in psychiatry and promotes a harmonization between research practice and clinical decision-making (19). This framework allows for transdiagnostic dimensions of psychopathology to be specifically studied and targeted and was well suited for the development of a new treatment modality for adolescent depression. Targeted constructs include the ones that have repeatedly been shown to be involved in adolescent depression, e.g., the first target is the construct of sustained threat and its associated limbic hyperreactivity (18). Subsequent targets are addressed progressively in a way that gives priority to the domains thought to be driving the psychopathology (18). The development process and a detailed description of the content of the different modules of TARA, as well as their relation to targeted constructs of the RDoC have been thoroughly elaborated in our previous publications in Frontiers’ journals (18, 20, 21). For example, the proposed working mechanism of TARA in relation to the first target of sustained threat and amygdala hyper-reactivity includes practices to increase vagal afference. Thus, in contrast to more cognitive top-down approaches here an initial bottom-up approach to emotional self-regulation is introduced before cognitive strategies are applied. Another contrast is that conventional talk therapy normally addresses depressive symptoms from an individual perspective whereas in TARA the focus in progressively changing from the individual to group processes in module three includes a more systemic perspective, suggesting that the depressive symptoms may also be a normal reaction to overwhelming life challenges and threats.

TARA has documented feasibility, acceptability, and preliminary efficacy in the treatment of depression in adolescents in the U.S., with significant improvement pre-post on both self and clinician rated depression severity, anxiety severity, sleep, psychological flexibility, and mindfulness skills (21). In Sweden, TARA has previously only been evaluated as an indicated prevention program for medical students with stress-related symptoms, where it was shown to be feasible, acceptable and, according to qualitative descriptions, empowering for the students (22). With the use of neuroimaging, it has furthermore been shown that postulated brain changes are achieved in response to TARA (23, 24).

All previously conducted studies of TARA have been single-armed and therefore not robust to various forms of bias (25). Pre-registration, as well as clinical studies outside or the U.S. are also lacking. Furthermore, the neuro-developmental trajectory of the cerebral cortex (26) suggests that adolescents and young adults share similarities that are rarely addressed in clinical trials, and very few studies have been conducted that cover the critical age-range of 15–22. To resolve remaining questions, we have planned and initiated a multicenter randomized controlled trial (RCT) (20), the pilot phase of which is reported here. Apart from an upcoming U.S. study that will use magnetic resonance imaging as primary outcome, the ISRCTN- and Clinicalrtrials.gov registers (as of February 2023) record no comparable recent nor ongoing trials anywhere in the world.

The objectives of the present study were to determine the feasibility of implementation of the trial protocol including data collection procedures at the different participating centers, as all but one had no experience in conducting clinical research, and to evaluate preliminary differences between pre- and post-treatment on the pre-specified primary and secondary outcome measures.

## 2. Materials and methods

For a more detailed description of all aspects of the methodology used in the present study and the TARA-RCT we refer to our open-access published study protocol (20) as well as the clinicaltrials.gov pre-registration with NCT-registration identifier: NCT04747340. Below we briefly review the methodological aspects central to the present study. We have followed the Consolidated Standards of Reporting Trials (CONSORT) guidelines (27), including extensions for non-pharmacological interventions (28) and pilot trials (29).

### 2.1. Recruitment

Participants were recruited from the Child and adolescent psychiatry (CAP) specialized outpatient academic unit and the outpatient community youth clinic (YC) in the university city Umeå, CAP and YC in Skellefteå and CAP in Örnsköldsvik, as well as in Sundsvall, all of which are cities in northern Sweden. Recruitment was done in three different ways: (1) by an assessment team at the time of an incoming referral to CAP, (2) by clinical staff recruiting participants either at their first visit, during ongoing standard treatment or from those wait-listed for treatment, and (3) by flyers posted in the clinics’ waiting rooms and at the student health clinic of the University of Umeå. Those who were interested in participation were contacted over the phone to get information about the study and an initial assessment of eligibility was performed. For adolescents who were not possible to reach over the phone, parents/legal guardians were contacted.

#### 2.1.1. Eligibility

Fifteen- to 22-year-old individuals that had been referred to one of the specialized CAP-canters or who were currently patients at any of the CAP or YC centers and had a diagnosis of either MDD or Persistent Depressive Disorder (PDD, previously Dysthymia) according to the Diagnostic and Statistical Manual of mental disorders−4th edition (DSM-IV), 5th edition (DSM-5), or the International Classification of Disease 10th edition (ICD-10) were eligible. The clinical diagnosis was validated with the Mini International Neuropsychiatric Interview for children and adolescents (30) version 6.0 for participants aged 15 to 17. The Mini International Neuropsychiatric Interview (31) version 7.0.0 was used for participants aged 18 to 22. These short and structured diagnostic interviews for psychiatric disorders are compatible with DSM IV-5 and ICD-10 and are useful tools for diagnostic screening both clinically (32) and in research (31). If results were inconclusive the baseline clinician rating on Children’s Depression Rating Scale—Revised (CDRS-R) (33) was used, and participants with scores above a cut-off of >40 were eligible. For participants in CAP centers that were below the age of 18 one parent/legal guardian had to be available and agree to participate in the first skills-training part of the online sessions.

Exclusion criteria have been described in detail elsewhere (20), briefly they included: (1) Having any severe psychiatric comorbidity or any severe psychiatric symptom(s)/behavioral problem(s) that may interfere with or hinder group participation. (2) Having a first-degree relative with bipolar disorder. (3) On-going trauma, neglect, abuse, domestic violence, or destabilizing legal processes. (4) Pregnancy. (5) Non-fluency in oral and written Swedish.

Psychiatric comorbidities such as attention deficit hyperactivity disorder (ADHD), any anxiety disorder, high-functioning autism spectrum disorder and mild to moderate eating disorders were allowed, and so was antidepressant medication at study start.

The trial was conducted in accordance with the ethical principles stated in the Declaration of Helsinki. Participant enrolment started in August 2020 and was completed in February 2022.

### 2.2. Data collection

Self-report scales were administered using an online platform and individual sign-in codes. Participants who missed assessments at any timepoint were sent automatic reminder(s) by email, then SMS, and lastly, if necessary, telephone contact was taken with the participant or a parent/legal guardian depending on the participant’s age. A brief questionnaire with only the primary and secondary outcome measures was available in case the participant did not manage to fill in the full set of questions, and in cases of extreme difficulties only the primary outcome was administered. In addition to the assessments reported below we followed a standard clinical procedure for routine depression related biochemical analysis of underlying somatic disease at T0. Three individuals with minor increases in homocysteine or low ferritin were referred to primary care for appropriate investigation, they were however still included in the study.

#### 2.2.1. Baseline measures for description of sample

The participants’ *sociodemographic background* was assessed using a brief self-made questionnaire. We used the *Childhood Trauma Questionnaire* to retroactively screen for adverse childhood events. This 28-item self-report measure assesses history of emotional abuse and neglect, physical abuse, and sexual abuse. The total scale adequately captures a broad dimension of childhood maltreatment, and higher scores indicate more severe maltreatment (34). Reliability and validity have been demonstrated in large samples, with psychiatrically referred groups reporting higher levels of abuse and neglect than non-clinical groups (34, 35).

#### 2.2.2. Feasibility and safety measures

The following measures were registered weekly: (1) Attendance rates. (2) The Outcome Rating Scale (36) was administered before each TARA-session. This is a measure of how the participant has been doing individually, in the family, socially, and overall, during the previous week. It was included to help detect any deterioration during treatment. (3) The Session Rating Scale (36) was administered after each TARA-session. This is a measure of working alliance, and the participants rate how much they felt listened to, how important the content and activities were to them, how much they liked the session, and their overall experience. The Outcome Rating Scale and Session Rating Scale each have four items on ten-centimeter visual analog scales, scores range from 0–10 on each item and 0–40 on the total scales, with higher scores indicating better functioning/experience (36). (4) A self-made scale was used to assess the facilitators adherence to the TARA-manual. And (5) A questionnaire on adverse events (AEs) occurring in the past week was administered before each TARA-session. AEs were defined as any occurrence that had required outpatient medical visits regardless of reason for contact and whether considered related to treatment or not. Examples were given of a range of things that would have constituted an AE and participants were asked to answer if any such event had occurred by yes/no. Affirmative answers were followed up with a free-text question on details of the event. Additionally, all AEs reported verbally by the participants or observed by the investigators, by TARA facilitators, by other health care providers, as well as those encountered at medical record analysis at the end of the study were recorded.

Other aspects of practical feasibility that were assessed included recruitment rates, participants’ need for extra support between sessions and equality of treatment effects across facilitators.

#### 2.2.3. Primary outcome measure

Primary outcome measure was self-reported depression symptom severity on the *Reynolds Adolescent Depression Scale 2nd edition* (RADS-2) (37) total raw-score at T1. RADS-2 has excellent psychometric properties and is validated in adolescents with depression (37), including in Swedish normative (38) and clinical (39) samples of adolescents and young adults. The scale has 30 items and four subscales measuring different dimensions of depression: Dysphoric Mood, Anhedonia/Negative Affect, Negative Self- Evaluation, and Somatic Complaints. Total raw-score range is 30 to 120 and higher scores indicate more severe depression.

#### 2.2.4. Secondary outcome measures

Secondary outcome measures were: (1) Clinician rating of depression symptom severity performed by trained clinicians on the *Children’s Depression Rating Scale - Revised* (CDRS-R) at T1. CDRS-R is a rating scale based on a semi-structured interview and provides an efficient way to monitor treatment response (33). Raw-score range is 17–113 and higher scores indicate more severe depression. In cases when the trained assessor was not a psychiatrist the assessments were recorded for quality control, this did not yield any adjustments to the scores. And (2) Self-rated anxiety-severity on the *Multidimensional Anxiety Scale for Children* (MASC) at T1. Anxiety disorders are highly comorbid with depression and MASC is considered the best normed and psychometrically strong self-report anxiety scale to use in adolescents (40, 41). Total raw score range is 0–117 and higher scores indicate more severe anxiety.

#### 2.2.5. Medical record data collection

We retroactively collected data from the medical records of all participants at T1, including information on new psychiatric diagnoses, treatments offered (psychological, pharmacological, inpatient care, and other), the number of and reasons for outpatient clinical visits, as well as any change of and termination of treatment. A descriptive summary of this information is reported.

### 2.3. Intervention

TARA is a non-pharmacological complex intervention that has previously been described in detail (18, 20, 21). In this study all participants received TARA according to the TARA intervention protocol formulated for the larger RCT (20) and there was no control condition. TARA was delivered to the first 12 participants face-to-face. Due to social distancing restrictions posed by the participating centers following the spread of the Coronavirus, subsequent groups were delivered online through a secure platform. The shift to online delivery was made after one additional TARA-group that had started face-to-face was forced to discontinue after four sessions as group activities were forcefully shut down. As this was an extreme event external to our control, the participants from that group were excluded from further follow-up and they are not included in the present sample. The only difference between the two delivery-formats of TARA was that with online delivery, participants in CAP centers were required to have one parent/legal guardian with them for the first skills-training part of the sessions. This was implemented to reduce the risk of non-attendance and drop-out due to the looser boundaries of this delivery-format. The national ethical review board approved these changes (registration number 2020-05734).

Briefly, TARA consisted of 12 weekly sessions, 90 min each, in groups of five to six participants. The first session aimed at creating a sense of safety by introducing the group members and facilitators; establishing clear guidelines; investigating attitudes, opinions, and previous experiences of group processes; explaining the format and introducing contemplative practices. During all sessions there was an explicit focus on transparency, authenticity, and mutual trust/safety to express subjective truths without being judged, punished, or excluded. Participants used yoga mats provided to them for the skills-training part of the session. In the online delivery version, the two facilitators were individually signed on to the online platform. For participants that did not have access to a computer or tablet, a tablet was provided that was returned at the end of the study. Facilitators “opened the circle” by ringing a bell and then briefly checked in with the participants. Yoga based movement followed, which consisted of a flow of simple positions synchronized with the breath. Next, participants were guided through gentle breathing practices and a short, guided meditation that was focused mainly on interoceptive and sensory awareness. After a short break there was a brief psychoeducational presentation followed by group assignments and discussions. Then there was time for feedback and questions regarding the home practice from the previous week and the participants were instructed in the practices for the coming week. Finally, the sessions ended with a “closing of the circle,” when the participants gathered their attention and had the opportunity to express any reflections or thoughts regarding the session.

Each TARA-session is described in detail in a facilitator-manual and all sessions for a given group were facilitated by the same two specifically TARA-trained facilitators, each with experience from clinical child and adolescent psychiatry or psychiatry and contemplative practice. Their professions included psychiatrists, resident physicians, clinical psychologists, researchers, and social workers. Facilitators were allocated to each trial group by the principal investigator and the directors of the different centers based on local availability. Fidelity to the TARA manual, both in terms of adherence to content and the process of delivery, was assessed formally with the use of a specific TARA-fidelity scale in four randomly selected sessions out of 12. This was done by one of two observers who passively attended each online session. Ongoing supervision and feedback were provided. The facilitators’ task during the sessions was not only to teach specific content, but also to model a collaborative, inclusive, non-judgmental, and supportive attitude. Home practice of TARA was encouraged and audio tracks with instructions on the different exercises were provided.

Facilitators observed adverse reactions in the TARA-groups closely and any deterioration of symptoms was discussed with the participant and their parents/legal guardians if appropriate.

Participants who did not show up for a session were contacted over the phone the following day to discuss the reasons for their non-attendance, to assess their status and safety, and to support their home practice. Outcome measures were collected also in cases of treatment non-adherence. Booster sessions including a summary of the previous session were offered if necessary to prevent drop-out, maximum *n* = 2 per participant, and the number of these is reported.

### 2.4. Statistical analysis

The planned analysis was outlined in detail in the pre-specified statistical analysis plan for the RCT (20). There was no missing item-level data on any of the outcome measures, and no imputation of missing scale-level data was performed. Analyses were performed by the investigators and a biostatistician using SPSS statistics (IBM Corp., Armonk, NY, United States) and R 4.2.2 (2022, R Core Team, Vienna, Austria). Significance-testing was two-tailed using a significance level of 0.05 and statistical uncertainties are expressed with 95% confidence intervals. Since statistical multiplicity does not arise when there is no opportunity to choose the most favorable outcome analyzed (42), our pre-specified secondary outcome-estimates are not corrected for multiple comparisons.

Participants who did not complete T0 assessments were considered external dropouts and were not included in the analysis. Participants who completed T0 but did not complete the treatment were considered internal dropouts and were included in the analysis performed according to intention-to-treat. All participants missing at T1 were contacted over the phone to clarify the causes of dropping out, some of them were not possible to reach. Participants who had dropped out of treatment were asked to still contribute with outcome data.

Standard measures were used for descriptive statistics, and feasibility-outcomes were analyzed and reported descriptively. The number of AEs are reported by (1) preferred term, (2) severity, and (3) their relationship as “definitely,” “probably,” “possibly,” and “unlikely” related to treatment.

The pre-specified mixed effects model included fixed effects for treatment allocation, RADS-2-score at T0, age group (dichotomous, 15–17 and 18–22 years old), sex and study center. In the present study all participants received TARA, and as there was only one TARA-group held at each center (two at one center) the two factors “treatment allocation” and “center” were not included in the present model. Primary and secondary outcome measures were analyzed using hierarchical mixed effects modeling to account for correlation within each treatment group, i.e., the clustering caused by partial nesting. The clustering effect was modeled using a random intercept for each intervention group, i.e., each group of participants who received TARA together.

The outcome differences between pre- and post-TARA were modelled using a longitudinal repeated measures approach with dummy variable representing time (0 for pre- and 1 for post TARA), with T0 (0) as reference time point. The correlation structure due to repeated measures and treatment group was modelled using random effects at participant level nested within intervention group. The models were adjusted for age group (dichotomous, 15–17 and 18–22 years old), and sex. In accordance with the analysis strategy of the RCT, compound symmetry was the most appropriate choice of correlation structure. The models were fitted using restricted maximum likelihood and assumptions were checked with residual plots. Secondary outcomes were analyzed in similar mixed effects models with fixed effects for the T0 score of each respective outcome instead of T0 score on RADS-2. For MASC the measurements did not converge due to larger dispersion within groups, and therefore the random effect was included only at the level of participant instead of intervention group.

Since this was a pilot study no sample size calculation was performed. The aim was to recruit six to eight participants to complete one TARA-group at each study center. After the shift to online treatment one center recruited participants to fill a second group for online treatment.

## 3. Results

### 3.1. Descriptive statistics

**Figure 1:**
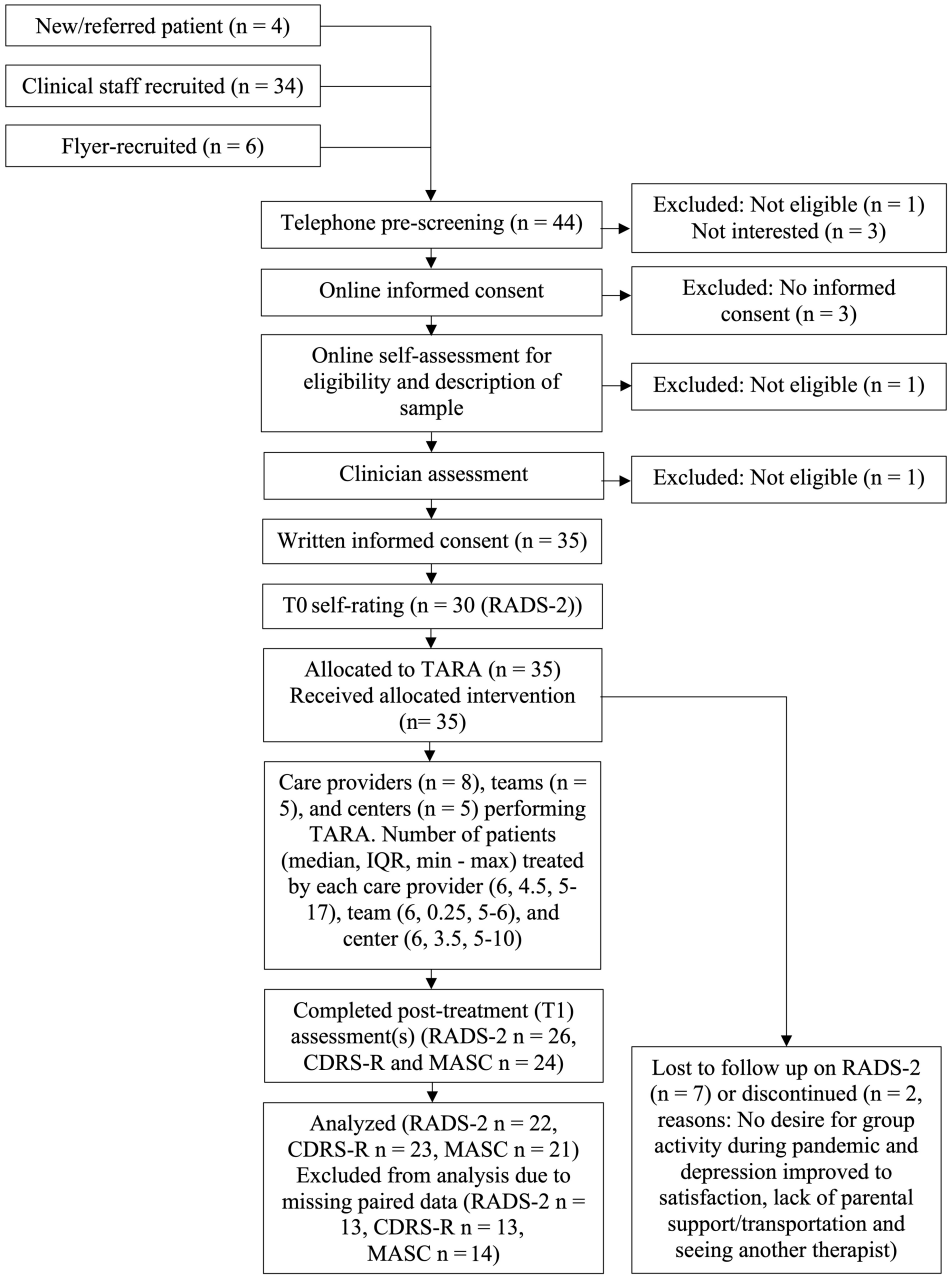
Flow of participants. CDRS-R, Children’s Depression Rating Scale—Revised; IQR, interquartile range; MASC, Multidimensional Anxiety Scale for Children; *n*, number of participants; RADS-2, Reynolds Adolescent Depression Scale 2nd edition; TARA, Training for Awareness, Resilience and Action.

In total, 35 participants (28 female, seven male) were allocated to six different TARA-groups, one at each of the five participating centers and two at the YC-Umeå center. The groups were led by 8 individual facilitators in different pairs. Please see Figure 1 for more detailed information in a CONSORT-diagram of the flow of participants.

The participant mean age was 17.7 years (SD 2.2, range 15–21). One participant who was assigned female at birth reported male gender. All participants were born in Sweden, as were both parents/legal guardians of 28 participants (80%). The remaining seven participants (20%) had one or two parents/legal guardians originally from other European, African, or Middle Eastern countries. The mean number of comorbid psychiatric diagnoses/participant was 3.7 (SD 1.5, range 1–7). All participants reported childhood trauma, with Childhood Trauma Questionnaire scores ranging from 29 to 101, mean 45.00 (SD 14.94). Please see Table 1 for more descriptive statistics of the sample.

**Table 1:** Descriptive statistics of the sample.

	*n* (%)
**Sex**	
Female	28 (80)
Male	7 (20)
**Recruited from**	
CAP-center Sundsvall	6 (17.1)
CAP-center Umeå	6 (17.1)
CAP-center Örnsköldsvik	5 (14.3)
YC-center Skellefteå	8 (22.9)
YC-center Umeå	10 (28.6)
**Recruitment method**	
New/referred patient	4 (11.4)
Clinical staff recruited	26 (74.3)
Flyer-recruited	5 (14.3)
**Living conditions**	
Living with both parents	13 (37.1)
Living with one parent	11 (31.4)
Living alone, with friend(s)/partner or others	11 (31.4)
**Working status of parent/caregiver (parent one/parent two)**	
Working fulltime	26 (74.3)/26 (74.3)
Working part-time	4 (11.4)/1 (2.9)
Studying fulltime	1 (2.9)/0 (0)
On sick leave	1 (2.9)/2 (5.7)
Unemployed	3 (8.6)/2 (5.7)
Unknown	0 (0)/4 (11.4)
**Feeling safe with current living situation**	
“Yes”	30 (85.7)
“No”	0 (0)
“Not sure”	5 (14.3)
**Smoking status**	
8–10 cigarettes daily	3 (8.6)
0–5 cigarettes monthly	1 (2.9)
Non-smoker	31 (88.6)
**ICD-10 diagnosis at baseline**	
Major depressive disorder	35 (100.0)
Dysthymia	15 (42.9)
Any anxiety disorder	31 (88.6)
ADHD	7 (20.0)
Psychosis (ever)	8 (22.86)
Obsessive–Compulsive disorder	6 (17.1)
PTSD	5 (14.3)
Tourette’s	2 (5.7)
Oppositional Defiant Disorder	2 (5.7)
Anorexia	2 (5.7)
Autism spectrum disorder	2 (5.7)
Alcohol- or substance use disorder	0 (0.0)
**TARA delivery format**	
TARA in real life	12 (34.3)
TARA online	23 (65.7)

ADHD, Attention Deficit Hyperactivity Disorder; CAP, Child and Adolescent Psychiatry; ICD-10, International Classification of Diseases 10th edition; *n*, number of participants; PTSD, Post Traumatic Stress Disorder; TARA, Training for Awareness, Resilience, and Action; YC, Youth Clinic.

The median duration of the current depressive episode was 120 days prior to T0 (IQR 232, range 10–2,223). Twenty-one participants (60%) had experienced one or more previous episodes of clinical depression. Eighteen participants (51%) had previously received psychological treatment(s) for depression, with a median number of visits of 8 (IQR 10.5, range 2–26) and a median treatment duration of 243 days (IQR 339.8, range 14–720). Most of the participants were prescribed psychopharmacological medication at T0 (*n* = 27, 77%). The median number of individual drugs per person was 2 (IQR 2, range 1–3). Please see Table 2 for details on prescribed psychopharmacological medication pre- and post-TARA.

**Table 2:** Prescribed psychopharmacological medication pre- and post TARA.

Type	Prescribed at T0, *n* (%)	Dose at T0, Median (IQR, range)	Prescribed at T1, *n* (%)	Dose at T1, Median (IQR, range)
**Daily**				
Sertraline	10 (28.6)	62.50 mg (62.50, 50.00–150.00)	9 (25.71)	75.00 mg (37.50, 50.00–150.00)
Fluoxetine	4 (11.43)	40.00 mg (45.00, 20.00–80.00)	4 (11.43)	40.00 mg (45.00, 20.00–80.00)
Duloxetine	1 (2.86)	60.00 mg	2 (5.71)	75.00 mg (30.00–120.00)
Venlafaxine	1 (2.86)	75.00 mg	2 (5.71)	225.00 mg (150.00–300)
Mirtazapine	1 (2.86)	15.00 mg	2 (5.71)	15.00 mg (0)
Melatonin	11 (31.43)	4.00 mg (6.00, 2.00–8.00)	12 (34.29)	5.00 mg (5.75, 2.00–10.00)
Escitalopram	0	-	1 (2.86)	10.00 mg
Methylphenidate	1 (2.86)	40.00 mg	0	-
**As needed**				
Clonazepam	1 (2.86)		1 (2.86)	
Codeine	1 (2.86)		1 (2.86)	
Hydroxyzine	9 (25.71)		11 (31.43)	
Promethazine	9 (25.71)		9 (25.71)	
Propiomazine	1 (2.86)		3 (8.57)	
Alimemazine	2 (5.71)		2 (5.71)	

*n* = number of participants.

The median time from T0 to starting TARA was 2 days (IQR 5.0, range −8 to 14), and the median time from T0 to T1 measurements was 107 days (IQR 19.0, range 92–156).

### 3.2. Outcome measures

#### 3.2.1. Feasibility and safety outcomes

**Figure 2:**
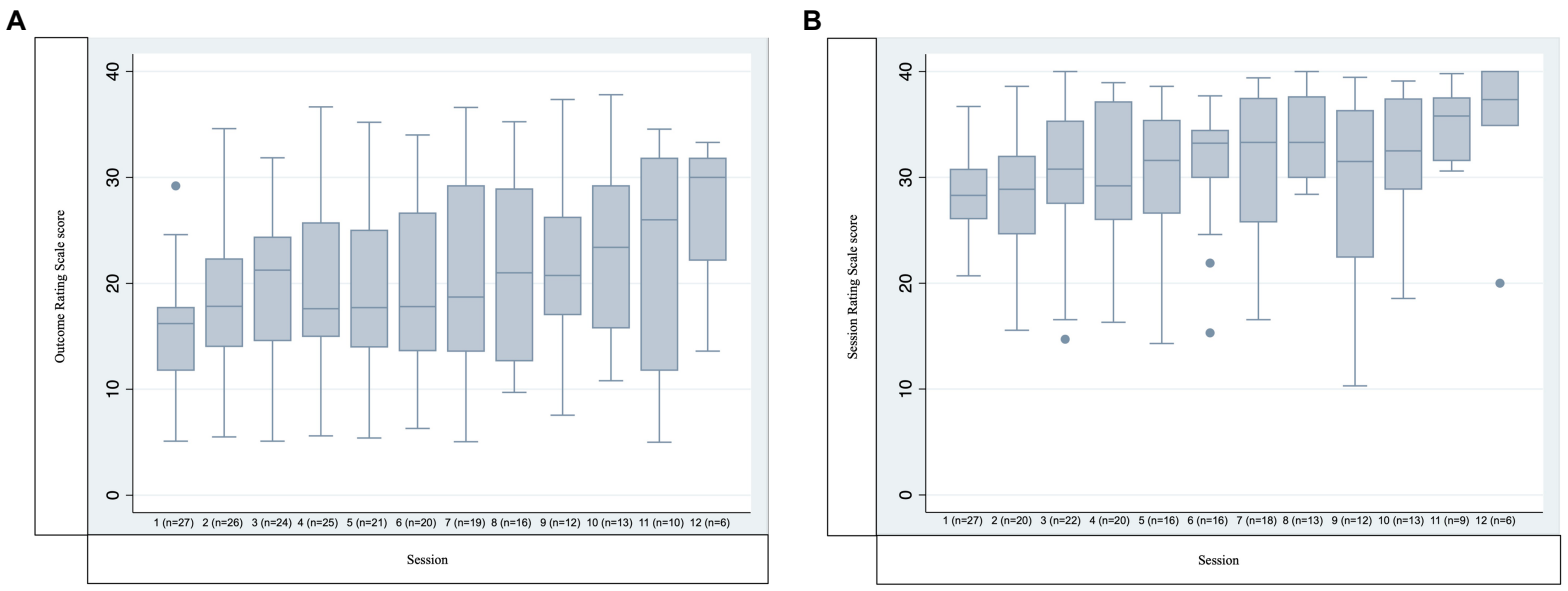
Outcome rating scale and session rating scale scores. **(A)** Outcome rating scale score. **(B)** Session rating scale score. *Y*-axis indicates the sum of all four items of each scale. *X*-axis indicates session number (and number of participants responding). Boxes represent median and interquartile range (IQR), whiskers represent the furthest point within 1.5 IQR of the nearer quartile.

Participants from the face-to-face-group that was discontinued by the center after four sessions due to the spread of the Coronavirus were excluded from further follow-up and analysis. The first online group (*n* = 6) was also discontinued halfway through treatment due to problems with the online platform, the facilitators’ lack of experience in online delivery, and participant non-attendance. These participants were still followed-up and are included in all analyses. The mean number of sessions attended per participant overall was 7.31 (SD 3.69, Range 1–12), and the mean number of sessions attended per accompanying parent/legal guardian was 8.27 (SD 3.20, Range 3–12). The participants’ attendance rate per session offered was 66.06% (SD 13.11, Range 37.93 (Session 9) – 88.57 (Session 1)), and the attendance rate for parents/legal guardians was 67.43% (SD 15.73, range 45.45–90.91). At three sessions one of the parents was present while the participant was absent. Two participants were given one booster session each, and one participant was given two booster sessions. Two brief supportive telephone calls were given to one of the participants to provide support around family issues. Outcome Rating Scale and Session Rating Scale total scores are presented in Figure 2.

For a descriptive summary of adverse events, please see Table 3.

**Table 3:** Summary of Adverse Events Between T0 and T1, Intention-To-Treat Population.

Nr. of events	Nr. of participants affected	Domain	Description	Severity	Relation to TARA
Mental health related issues		Profession or type of complaint			
13	8	Psychiatrist	Dose-adjustment, change, or termination of antidepressant	Mild	Possibly
1	1	Psychiatrist	Assessment of suicidal communication	Moderate	Possibly
1	1	Psychiatrist	Assessment of depressive symptoms	Moderate	Possibly
4	3	Psychologist	Diagnostic assessment	Mild	Unlikely
4	2	Psychologist	Supportive talk therapy	Mild	Possibly
1	1	Psychologist	Neuropsychiatric assessment	Mild	Unlikely
1	1	Psychologist	Assessment of anxiety and mild self-harm	Mild	Possibly
1	1	Psychologist	Assessment of worry and insomnia	Mild	Possibly
1	1	Occupational therapist	Consultation regarding ADL	Mild	Possibly
Somatic complaints		Medical assessment or treatment*			
3	2	Gastrointestinal	Stomach pain not otherwise specified	Mild	Unlikely
3	3	Gynecological	Gynecological complaint	Two mild, one moderate	Unlikely
2	1	Neurological	Neurorehabilitation	Mild	Unlikely
3	3	Musculoskeletal	Traumatic injuries	Mild	Unlikely
3	3	Respiratory	Respiratory problems	Mild	Unlikely
2	2	Ophthalmological	Conjunctivitis	Mild	Unlikely
3	3	Other	Other minor somatic complaints	Mild	Unlikely
2	2	Musculoskeletal (physiotherapist)	Assessment of pain	Mild	Possibly

*All events where profession is not specified were visits to medical doctors. ADL = Activities of Daily living.

Weekly assessments of facilitators’ fidelity to the TARA manual revealed no major deviations, neither in terms of adherence to content nor in the process of delivery. For a discussion on other aspects of feasibility, please see “*Discussion*.”

#### 3.2.2. Primary and secondary outcome measures

In the whole sample trends toward improvement were seen across all primary and secondary outcome measures, both on total scores and all subscale scores. For detailed descriptive pre-post data, please see Table 4.

**Table 4:** Descriptive pre-post data on primary and secondary outcome measures including subscales, in test–retest samples presented by sex and age categories, with subgroups combined, as well as for the whole sample.

Outcomes and subscales	Test–retest samples, Mean (SD)										Whole sample, Mean (SD)	
	Females		Males		Age < 18 years		Age ≥ 18 years		All combined			
	T0	T1	T0	T1	T0	T1	T0	T1	T0	T1	T0	T1
RADS-2	*n* = 17		*n* = 5		*n* = 10		*n* = 12		*n* = 22		*n* = 30	*n* = 26
Total score	80.18 (12.68)	78.24 (19.52)	86.40 (9.40)	84.60 (11.30)	82.40 (14.39)	86.70 (16.89)	80.92 (10.44)	73.83 (17.31)	81.59 (12.10)	79.68 (17.95)	84.63 (12.52)	78.81 (17.20)
Dysphoric mood	22.71 (3.41)	22.53 (4.47)	21.60 (3.44)	21.00 (5.00)	21.80 (3.58)	22.80 (4.98)	23.00 (3.22)	21.67 (4.25)	22.45 (3.36)	22.18 (4.52)	23.30 (3.43)	22.23 (4.35)
Anhedonia/negative affect	16.94 (5.33)	15.41 (6.10)	18.20 (3.96)	19.20 (4.21)	17.60 (5.80)	18.50 (6.38)	16.92 (4.46)	14.42 (4.91)	17.23 (4.99)	16.27 (5.87)	17.50 (4.83)	16.04 (5.57)
Negative self-evaluation	20.47 (4.61)	20.53 (6.66)	23.60 (3.51)	23.20 (4.14)	21.70 (4.85)	24.00 (4.57)	20.75 (4.37)	18.75 (6.52)	21.18 (4.51)	21.14 (6.20)	22.50 (4.85)	20.42 (6.01)
Somatic complaints	20.06 (3.36)	19.76 (3.93)	23.00 (1.87)	21.20 (4.32)	21.30 (3.83)	21.40 (4.03)	20.25 (2.86)	19.00 (3.72)	20.73 (3.30)	20.09 (3.96)	21.33 (3.24)	20.12 (3.78)
CDRS-R	*n* = 19		*n* = 4		*n* = 13		*n* = 10		*n* = 23		*n* = 34	*n* = 24
Total score	57.13 (13.06)	46.37 (12.31)	54.75 (5.84)	55.25 (8.77)	57.08 (9.91)	51.46 (10.75)	56.25 (14.95)	43.30 (12.71)	56.72 (12.05)	47.91 (12.09)	58.10 (13.22)	47.25 (12.27)
MASC	*n* = 16		*n* = 5		*n* = 9		*n* = 12		*n* = 21		*n* = 30	*n* = 24
Total score	53.19 (19.97)	55.81 (19.67)	59.20 (27.12)	59.80 (17.47)	50.33 (25.09)	61.56 (23.65)	57.83 (18.44)	53.17 (14.31)	54.62 (21.29)	56.76 (18.83)	58.23 (21.42)	56.67 (17.71)
Physical symptoms	16.31 (7.00)	17.00 (7.16)	20.00 (9.57)	20.20 (6.30)	15.00 (9.26)	18.67 (7.45)	18.83 (5.97)	17.08 (6.82)	17.19 (7.59)	17.76 (6.96)	18.60 (8.19)	17.54 (6.56)
Harm avoidance	16.06 (5.23)	16.63 (4.51)	16.60 (7.86)	15.00 (6.44)	15.44 (5.88)	16.00 (5.92)	16.75 (5.83)	16.42 (4.27)	16.19 (5.74)	16.24 (4.91)	16.63 (5.35)	16.29 (4.73)
Social anxiety	13.81 (7.24)	14.44 (7.20)	15.80 (4.76)	17.40 (4.67)	13.33 (7.70)	17.44 (6.88)	15.00 (6.06)	13.42 (6.30)	14.29 (6.68)	15.14 (6.70)	15.57 (6.76)	15.50 (6.37)
Separation anxiety	7.00 (5.38)	7.75 (6.70)	6.80 (6.42)	7.20 (3.96)	6.56 (5.61)	9.44 (6.62)	7.25 (5.59)	6.25 (5.51)	6.95 (5.47)	7.62 (6.07)	7.43 (5.18)	7.33 (5.80)

CDRS-R, Children’s Depression Rating Scale – Revised; MASC, Multidimensional Anxiety Scale for Children; *n*, number of participants; RADS-2, Reynolds Adolescent Depression Scale 2nd edition.

For the primary outcome RADS-2 the Pearson test–retest correlation coefficient was 0.70 and the Intraclass Correlation Coefficient was 0.09. The primary outcome mixed-model included 22 participants and the adjusted pre-post mean difference in RADS-2 total score was −3.26, 95% CI –8.35 to 1.83; *p* = 0.20. Secondary outcome models included 22 and 21 participants on CDRS-R and MASC, respectively. The adjusted pre-post mean difference in CDRS-score was −9.99, 95% CI –14.76 to −5.22; *p* < 0.001, and the adjusted pre-post mean difference in MASC total score was 1.98, 95% CI –0.96 to 4.91; *p* = 0.18. Full model outputs are presented in Supplementary material S1.

### 3.3. Medical record analysis

The medical records of all 35 participants were analyzed. There were no suicide attempts and no participant had been admitted to psychiatric inpatient care during the study. Outpatient visits to medical personnel regardless of profession and reason for contact were considered adverse events and are reported in Table 3.

For a summary of prescribed psychopharmacological medication at T0 and T1 please see Table 2. No cases of medication non-adherence were registered other than one participant not adhering to prescribed Melatonin. Psychiatric diagnoses at T0 are reported in Table 1. Additional psychiatric diagnoses that had been given to participants between T0 and T1 included dysthymia, unspecified eating disorder, unspecified anxiety-disorder, and neurasthenia (all *n* = 1), as well as ADHD and mixed anxiety and depressive disorder (both *n* = 2).

Three participants received individual CBT concurrent with TARA, median number of sessions offered and attended was 3 (range 3–4).

The medical records of the nine participants without T1 data on RADS-2 indicated that only three of them had any subsequent visit for psychiatric problems registered, two for depression and one for ADHD. Two had no contact with healthcare at all and the other four had somatic problems that are reported in Table 3.

## 4. Discussion

This single-arm pilot study constitutes the first step in a large systematic evaluation of TARA in the treatment of adolescents and young adults with clinical depression in a Swedish context. We are the first to present data supporting the feasibility of delivering TARA, including online, to a mixed clinical sample and demonstrate positive group-level trends on pre-specified primary and secondary outcome measures.

Our trial results echo those of previous studies of TARA face-to-face in the U.S., including the only study of TARA performed in a clinical sample (21). Compared to the participants in the U.S. study our sample scored higher at T0 on measures of depression (84.63 compared to 78.54 on RADS-2, and 58.10 compared to 46.88 on CDRS-R) and lower on anxiety scores (58.23 compared to 63.92 on MASC), although scoring differences may not necessarily reflect true differences across languages. High T0 self-rating scores on all outcome measures and childhood trauma, extensive and complex comorbidity as well as prescribed polypharmacy indicate that the current sample was a representative sample of clinically depressed individuals.

In terms of feasibility, the pre-specified recruitment methods were satisfactory, although males were harder to recruit than females and this of course affects external validity. Future recruitment rates may change with the addition of randomized treatment allocation. Attendance rates were negatively affected by the first online group that had to be discontinued due to participant non-attendance. The shift from treatment face-to-face to treatment online required a significant readjustment for the facilitators and study personnel and in the groups that followed the first, participants were retained satisfactorily. Apart from the discontinued group it was logistically feasible to recruit centers and facilitators with little or no previous background in clinical research and have them perform TARA, including online. The Swedish clinical specialist and non-specialist settings constitute very different contexts from the ones in previous studies of TARA, such as for example, school wellness centers, which adds confidence in the universal applicability of the intervention. No serious adverse events were observed, and no adverse event was classified as probably or definitely related to TARA.

On the other hand, Session Rating Scale scores were generally below 36 which is the suggested threshold for treatment alliance (43). This was possibly due to the online format and disease severity of the sample. Scores increased over the course of treatment, and this may be due to selective retention of participants that were satisfied with the intervention. It is also possible that the treatment progression improved the ratings over time. Data was furthermore collected in several ways, and the proportion of missing data indicates that the feasibility of our data-collection procedures needs to be addressed. This is unfortunately common in samples like the present (11, 44, 45), and we have developed a strategy to improve this in the RCT that includes coordination of self-rating and clinician rating.

Some of the present results align clearly with what was expected *a priori*. For RADS-2 the Pearson test–retest correlation coefficient was 0.70 (0.7 was expected), and the standard deviations were 12.52 at T0 and 17.20 at T1 (15 was expected). On the other hand, the Intraclass Correlation Coefficient was 0.09 (0.3 was expected). This indicates that the group-effect might be lower than expected, although a larger number of groups would be needed for firm conclusions.

Furthermore, both the effect-size and participant attendance/retention rates were also lower than expected. We propose several potential explanations for these results. First, although RADS-2 scores are not directly comparable across contexts, we included participants that were more severely depressed than in previous samples. Clinical observations indicated that the individuals with the highest T0 scores on depression severity were indeed the ones who had the largest difficulties to assimilate the concepts and practices of the intervention. Four of the six participants with the highest T0 ratings were also lost to follow-up at T1. The rates of medication were furthermore high and given the content and mechanisms of TARA one may hypothesize that emotional blunting, a symptom reported by nearly half of all patients on antidepressants (46), is particularly undesirable for TARA to be effective. It is possible that including a less severely ill clinical sample with less medication would have yielded better results both on retention and on treatment effect.

Second, the forced transition to online delivery, although appreciated by some, may have affected both facilitators and participants as well as the quality of connection and relationship between them. Heterogeneous use and adherence has been observed in digital mental health interventions (47–50) and face-to-face interventions enable more opportunities to build mutual trust and treatment alliance, which has been shown to mediate therapeutic effects (51). The online delivery format of TARA may have reduced participant engagement compared to the face-to-face format and relying solely on online communication may have hindered knowledge transfer, alliance, and sense of community.

Third, this study was performed during the peak of the Coronavirus pandemic, a time when depressive symptoms worsened in the present age range, particularly in females (52). Dramatic disruptions were seen in the everyday life of young individuals (53), with pervasive social isolation, missed milestones, distance schooling, quarantine, increased family stress, and decreased interactions with buffering support such as peers and teachers. Globally the rates of clinically elevated depression and anxiety symptoms have doubled compared to pre-pandemic estimates (54), and any positive effects of TARA may therefore have been masked by the negative effects of the pandemic.

In the present study TARA did not improve self-rated depression severity on the primary outcome RADS-2, a statistically significant improvement was however seen on the secondary outcome measure of clinician-rated depression severity. As the sample size was determined based on our aim to evaluate feasibility and not effectiveness, this is interpreted as a positive finding. The trends were stronger on the measures of depression compared to anxiety, and this was expected since TARA was designed to primarily treat depression. The lack of convergence on MASC is not expected in the RCT, as the sample size will be larger. Trending differences between males and females and between participants above and below 18 years of age indicated a more beneficial outcome in the older age group and in the female participants, which is in line with previous studies of outcome predictors and moderators in adolescent depression trials (55). None of the factors was statistically significant in the RADS-2 mixed model, and more formal subgroup comparisons were not performed for reasons of statistical multiplicity.

A close to significant positive correlation was found between childhood trauma measured with CTQ (log-transformed due to significant outliers) and RADS-2-scores at T0 (*r* = 0.33, *p* = 0.09), a finding consistently shown in previous studies (21, 56, 57). No significant correlation was seen between CTQ-score at T0 and RADS-2 change between T0 and T1 (*r* = 0.27, *p* = 0.22). This replicates previous findings (21) suggesting that the effects of TARA may be independent of trauma experience.

As a result of this pilot trial, we have made several important improvements to the design of the RCT: (1) Booster sessions are offered right away if a participant misses a session, still with a maximum number of two, with the hope that this will reduce attrition. (2) Recruitment is performed region-wide in regions where different centers share common administration. This technically reduces the number of centers and improves recruitment-rates further, as participants from (previously) different centers now are treated in the same groups. (3) The eligibility criteria have been extended to enable inclusion of participants from the year they turn 15 instead of from the day they turn 15, and the cut-off for CDRS-R recruitment has been reduced from 40 to 35. And (4) The parents/legal guardians that are participating in the CAP groups are offered three additional online meetings, in conjunction with the first, the sixth and 12th TARA-sessions. This forum was requested in parental interviews and will be offered for them to better support their child between sessions. Parental participation even in the absence of the participant occurred a few times and is interpreted as a sign that they too found the training important and worthwhile.

Weaknesses of the present study include the single-arm design, the presence of parallel treatments in addition to TARA, the relatively small sample size, and the amount of missing data. To have piloted randomization to standard treatment or TARA would have allowed for better prediction of recruitment and retention rates in the RCT, yet we decided not to do so to minimize time and resource consumption. In this study, the magnitude of the effects and in the case of MASC even the direction of the effect changed between the whole sample and the sample with data at both time points. This indicates non-random missingness and reduces interpretability. Another aspect that affects the interpretation is the forced switch from face-to-face to online delivery-format. The relevant CONSORT statement endorses methodological changes to pilot study designs even after the trial commencement (29) and no significant effects of delivery format were seen, although power was lacking for subgroup analysis. Different raters performed CDRS-R at T0 and T1, and this as well as rater non-blinding may also have confounded positive effects. In the RCT the same rater will perform both T0 and follow-up ratings for a given participant, and the raters will be blind to treatment allocation. Also, a general difficulty of studying the present age range is the lack of outcome measures that have been psychometrically evaluated in both adolescents and young adults. RADS-2 has been validated in the full age range of the present study (39), the properties of CDRS-R and MASC are however unknown in the oldest adults of the present study. The follow-up time was furthermore brief, this should however not bias results in favor of TARA as positive effects have been shown to increase over time in a previous study (21).

Strengths of the present study include the pre-registration and detailed pre-specification of methods in a publicly available study protocol. Deviations from these are reported explicitly. The inclusion of medical record analysis of adverse events does add credibility to the feasibility analysis, and parallel treatments are reported in detail. Half of the participants were recruited from specialist care and the other half from primary care, and although it was not meaningful to compare the effects, indicators of contextual generalizability were seen. This is also the first clinical study of TARA performed outside of the U.S., which adds information related to the generalizability across populations. The present age-range is also well in line with recent calls for research spanning the critical transition from adolescence into young adulthood (58, 59).

In summary, considering the predicted increase in the global burden of depression, it is desirable and viable to deliver evidence-based interventions to young individuals online. We conclude that TARA is feasible and safe in adolescents and young adults with depression, and as preliminary signs of effectiveness were seen the planned and initiated RCT will constitute an important step forward for research on depression.

## Data availability statement

The raw data supporting the conclusions of this article will be made available by the authors, without undue reservation.

## Ethics statement

The studies involving human participants were reviewed and approved by the Regional ethical review board in Umeå (registration number 2018-221-31) and the Swedish national ethical review board (registration numbers 2020-05734, 2021-06418, and 2022-04979). Written informed consent to participate in this study was provided by the participants. For participants between 15 and 17 years of age both parents/legal guardians also provided written informed consent.

## Author contributions

EE and EH performed the conception and design of the study. LR, RS, MS, and CS contributed valuable strategical input on scientific and practical issues. EE and GG conceived and performed data analysis. MS and EE performed medical record analysis. EE, EH, and LR drafted the manuscript. All authors approved the final manuscript.

## Funding

This study was funded by the Swedish Research Council (2021-02257), intramural funding from Umeå University (970831) the County Council of the Region Västerbotten (RV-939199, RV-967045, RV-969368, RV-941585, and RV-932919), the County Council of the Region Västernorrland, municipality of Örnsköldsvik and the Kempe foundation (LVNFOU933598), Lars Jacob Boëthius foundation, the Oskar foundation, and the Swedish society of medicine (SLS-935854). The funders had no role in the design, methods, subject recruitment, data collection, analysis, or preparation of the manuscript.

## Conflict of interest

The authors declare that the research was conducted in the absence of any commercial or financial relationships that could be construed as a potential conflict of interest.

## Publisher’s note

All claims expressed in this article are solely those of the authors and do not necessarily represent those of their affiliated organizations, or those of the publisher, the editors and the reviewers. Any product that may be evaluated in this article, or claim that may be made by its manufacturer, is not guaranteed or endorsed by the publisher.

## Abbreviations

ADHD: Attention Deficit Hyperactivity Disorder
AE: Adverse Event
CAP: Child and Adolescent Psychiatry
CBT: Cognitive Behavioral Therapy
CDRS-R: Children’s Depression Rating Scale-Revised
CONSORT: Consolidated Standards Of Reporting Trials
DSM-IV: Diagnostic and Statistical Manual of mental disorders−4th edition
DSM-5: Diagnostic and Statistical Manual of mental disorders−5th edition
ICD-10: International Classification of Disease 10th edition
MASC: Multidimensional Anxiety Scale for Children
MDD: Major Depressive Disorder
PDD: Persistent Depressive Disorder
PTSD: Post Traumatic Stress Disorder
RADS-2: Reynolds Adolescent Depression scale 2nd edition
RCT: Randomized Controlled Trial
RDoC: Research Domain Criteria of the National Institute of Mental Health
SSRI: Selective Serotonin Reuptake Inhibitor
TARA: Training for Awareness, Resilience, and Action
YC: Youth Clinic
