# Supplementary material for: Clinical effectiveness of training for awareness, resilience, and action for adolescents and young adults with depression: The pilot phase of a multicenter randomized controlled trial
Source: Front Psychiatry. 2023 Mar 31;14:1130035. doi: 10.3389/fpsyt.2023.1130035 (PMC10102586; doi:10.3389/fpsyt.2023.1130035)
Supplement: Supplementary file 1 [file Data_Sheet_1.docx]

*Supplementary material 1: Fixed effect estimates of linear mixed effects regression models, and false discovery rate corrected p-values.*

RADS-2

RADS-2 ~ t + age + sex + (1 | Group/subject_id)

Parameter | Coefficient | SE | 95% CI | t(49) | p

-------------------------------------------------------------------

(Intercept) | 89.49 | 3.80 | [ 81.84, 97.13] | 23.52 | < .001

t | -3.26 | 2.53 | [ -8.35, 1.83] | -1.29 | 0.204

age [18-22] | -11.41 | 4.81 | [-21.07, -1.74] | -2.37 | 0.022

sex [Male] | -0.37 | 5.73 | [-11.89, 11.15] | -0.06 | 0.949

CDRS-R

CDRS-R ~ t + age + sex + (1 | Group/subject_id)

Parameter | Coefficient | SE | 95% CI | t(51) | p

-------------------------------------------------------------------

(Intercept) | 62.27 | 4.13 | [ 53.98, 70.55] | 15.08 | < .001

t | -9.99 | 2.38 | [-14.76, -5.22] | -4.20 | < .001

age [18-22] | -9.53 | 4.75 | [-19.07, 0.01] | -2.01 | 0.050

sex [Male] | -1.92 | 4.64 | [-11.24, 7.40] | -0.41 | 0.681

MASC

MASC ~ t + age + sex + (1 | Group/subject_id)

Parameter | Coefficient | SE | 95% CI | t | df | p

------------------------------------------------------------------------

(Intercept) | 63.74 | 5.26 | [ 53.27, 74.22] | 12.12 | 74 | < .001

t | 1.98 | 1.47 | [ -0.96, 4.91] | 1.34 | 74 | 0.183

age [18-22] | -9.36 | 6.79 | [-23.35, 4.63] | -1.38 | 25 | 0.181

sex [Male] | -6.35 | 8.30 | [-23.43, 10.74] | -0.76 | 25 | 0.451

False discovery rate corrected p-values

RADS 0,204

CDRS-R <0,001

MASC 0,204

Note: CDRS-R = Children’s Depression Rating Scale – Revised, MASC = Multidimensional Anxiety Scale for Children, RADS-2 = Reynolds Adolescent Depression Scale 2nd edition.
